# Intravascular imaging-guided versus angiography-guided percutaneous coronary intervention in multivessel coronary artery disease: a systematic review, meta-analysis, and trial sequential analysis of randomized controlled trials

**DOI:** 10.1186/s12872-026-06135-7

**Published:** 2026-06-15

**Authors:** Ahmed Adel Mohamed, Asmaa Magdy Elhefnawy, Abdalrahman Salah Shehata, Sara Khalid, Abdelrahman Mostafa, Abdullah Ali, Baher Ibrahim, Tasneem Zanati Saeed, Mohammed Dibas

**Affiliations:** 1 Faculty of Medicine, Zagazig University, Zagazig, Egypt; 2 Faculty of Medicine, Qena University, Qena, Egypt; 3 Faculty of Medicine, Helwan University, Cairo, Egypt; 4 Faculty of Medicine, Alexandria University, Alexandria, Egypt; 5 Faculty of Medicine, Faqous Branch, Zagazig University, Zagazig, Egypt; 6 Faculty of Medicine, Assiut University, Assiut, Egypt; 7 Department of Medicine, Faculty of Medicine and Health Sciences, An-Najah National University, PO. Box 7, Nablus, Palestine

**Keywords:** Coronary artery disease, Intravascular imaging, Intravascular ultrasound, Major adverse cardiovascular events, Multivessel coronary artery disease, Optical coherence tomography, Percutaneous coronary intervention

## Abstract

**Background:**

Percutaneous coronary intervention (PCI) in patients with multivessel coronary artery disease is associated with increased anatomical complexity and adverse cardiovascular outcomes. Conventional angiography provides limited assessment of vessel structure and plaque characteristics, whereas intravascular imaging with intravascular ultrasound (IVUS) or optical coherence tomography (OCT) may improve procedural guidance. However, the clinical impact of imaging-guided PCI in this population remains uncertain.

**Methods:**

We conducted a systematic review and meta-analysis of randomized controlled trials comparing intravascular imaging-guided versus angiography-guided PCI in patients with multivessel coronary artery disease regarding major adverse cardiovascular events (MACE). Major databases were searched through December 2025. Outcomes were pooled as risk ratios (RRs) with 95% confidence intervals (CIs) using random-effects models. Trial sequential analysis (TSA) and an exploratory network meta-analysis (NMA) were performed.

**Results:**

Five randomized trials, including 3,023 patients, were analyzed. Imaging-guided PCI was associated with a significant reduction in MACE compared with angiography-guided PCI (RR 0.58, 95% CI 0.46 to 0.74; *p* < 0.0001; I^2^ = 9.8%). In subgroup analyses by imaging modality, IVUS-guided PCI was associated with a reduction in MACE (RR 0.53, 95% CI 0.39 to 0.73; *p* < 0.0001; I^2^ = 0%), as was OCT-guided PCI (RR 0.49, 95% CI 0.28 to 0.87; *p* = 0.014; I^2^ = 24.3%). TSA provided supportive evidence for a beneficial effect of imaging-guided PCI. NMA suggested that both IVUS and OCT were associated with improved outcomes compared with angiography, with no significant difference between the two imaging modalities.

**Conclusion:**

Intravascular imaging-guided PCI is associated with a reduction in MACE in patients with multivessel coronary artery disease. These findings support the clinical value of intravascular imaging in this population, with similar effects observed across imaging modalities.

**Supplementary Information:**

The online version contains supplementary material available at 10.1186/s12872-026-06135-7.

## Introduction

Percutaneous coronary intervention (PCI) is regarded as a cornerstone therapy in patients with symptomatic coronary artery disease [1]. Among patients with multivessel coronary artery disease, PCI is frequently selected as the revascularization strategy in contemporary clinical practice, as supported by large registry data and international revascularization guidelines [2–4]. However, PCI in patients with multivessel coronary artery disease remains technically challenging and, particularly among those unsuitable for or declining coronary artery bypass grafting, has been associated with higher rates of adverse cardiovascular events compared with less complex coronary disease [5].

Conventional PCI is mainly guided by coronary angiography, which offers a two-dimensional view of the coronary lumen. While this approach is widely used, it does not allow direct visualization of the vessel wall or a detailed assessment of plaque burden and morphology, especially in patients with multivessel coronary artery disease, where lesions are often more complex, and atherosclerosis is frequently diffuse, making more comprehensive anatomical and plaque evaluation helpful for optimizing revascularization strategies [4, 6, 7].

Over recent years, intracoronary imaging modalities have been progressively adopted into PCI practice to address these limitations. Intravascular ultrasound (IVUS) permits evaluation of vessel dimensions and plaque burden with sufficient tissue penetration, aiding appropriate stent sizing and procedural optimization [8]. Optical coherence tomography (OCT), owing to its high spatial resolution, offers detailed visualization of plaque features and stent-related complications, enabling precise procedural guidance [9, 10]. These imaging modalities have shown potential advantages in improving procedural results and lowering adverse cardiovascular events when compared with angiography-guided PCI [11–14].

In contemporary interventional cardiology practice, the use of intravascular imaging has been steadily increasing, driven by accumulating evidence supporting its role in optimizing stent deployment and improving procedural outcomes [7, 15]. This growing adoption is reflected in recent guideline recommendations, which recognize intravascular imaging as a valuable adjunct, particularly in complex coronary anatomies [16]. As evidence continues to evolve, intravascular imaging may play an expanding role in shaping future guideline-directed PCI strategies, especially in high-risk populations such as patients with multivessel coronary artery disease.

Despite these advances in imaging-guided PCI, evidence specifically evaluating its role in patients with multivessel coronary artery disease remains limited. Most available studies and systematic reviews have assessed heterogeneous patient populations or concentrated on selected lesion types, with relatively limited analyses dedicated to multivessel disease. As a result, the effect of imaging-guided PCI on clinical outcomes in this high-risk population remains limited.

Accordingly, this systematic review and meta-analysis aim to compare imaging-guided PCI using IVUS or OCT with conventional angiography-guided PCI in patients with multivessel coronary artery disease, with particular emphasis on clinical major adverse cardiovascular events (MACE). The randomized trials included in this analysis and their corresponding acronyms are summarized in Table S1.

## Methods

### Study design and registration

The meta-analysis was conducted in accordance with the PRISMA guidelines and the Cochrane Handbook of Systematic Reviews and Meta-Analysis [17, 18]. This review was registered in PROSPERO (CRD: 420251273489) [19].

### Eligibility criteria

Only randomized controlled trials were eligible for inclusion based on the following criteria: (1) Population: Patients with multivessel coronary artery disease undergoing PCI. (2) Intervention: PCI guided by intracoronary imaging modalities. Included imaging modalities are either OCT, IVUS, or studies containing both. (3) Comparator: Conventional angiography-guided PCI. (4) Outcome: MACE. Non-randomized controlled trials and studies that did not report outcomes of interest, as well as cross-sectional studies, case reports, case series, and reviews, were excluded.

### Search strategy

We searched PubMed, Scopus, Web of Science, and Cochrane CENTRAL. The search strategy combined Medical Subject Headings (MeSH) terms and free-text keywords related to the population, intervention, and study design, and is presented in Table S2. PubMed key search terms included: (“Intravascular Imaging” OR “coronary imaging” OR “vascular imaging” OR “intravascular ultrasound” OR “IVUS” OR “vascular ultrasound” OR “IVUS-guided PCI” OR “optical coherence tomography” OR “OCT” OR “angioscopy” OR “intravascular fluoroscopy”) AND (“coronary angiography” OR “cardiac angiography” OR “angiography” OR “coronary arteriography” OR “cardiac catheterization”) AND (“multivessel disease” OR “multivessel coronary disease” OR “multiple vessel disease” OR “three-vessel disease” OR “two-vessel disease” OR “complex lesion” OR “diffuse coronary disease” OR “complex coronary lesions”). The PubMed strategy was adapted for the other databases. There were no restrictions on language or publication date. Databases were searched from inception to December 16, 2025. Moreover, the reference lists of included studies and pertinent systematic reviews were carefully reviewed to identify any additional relevant studies.

### Study selection and data extraction

Search results were imported into Rayyan [20], and duplicate records were removed. Independent reviewers (A.M. and A.A.) screened titles and abstracts in accordance with the eligibility criteria. Potentially relevant studies underwent full-text review. Any discrepancies between the reviewers were resolved through consensus or consultation with a third reviewer (A.A.M.).

A standardized, pre-piloted data extraction form was used to systematically collect information from all included studies. Extracted data encompassed study characteristics (trial name, year of publication, study design, and country), patient characteristics (sample size, age, sex, clinical presentation, and prevalence of multivessel coronary artery disease), procedural characteristics (type of intravascular imaging used [IVUS or OCT], comparator strategy, PCI characteristics including multivessel PCI where applicable, and stent type), follow-up duration, and MACE data. All data were extracted directly from the published manuscripts and their supplementary materials. Definitions of multivessel coronary artery disease were extracted as reported in the individual trials and are summarized in the summary and baseline table (Table 1). However, variability in definitions across studies and incomplete reporting in some trials were noted. For outcome data extraction, multivessel coronary artery disease was identified based on trial-reported definitions. Subgroup data were extracted from published reports when explicitly reported. In the included trials, subgroup analyses on multi-vessel PCI population were generally reported as prespecified in the primary publications; however, detailed confirmation of prespecification was not consistently available in publicly accessible trial protocols. When an anatomical definition was not explicitly provided, data from patients undergoing multivessel PCI were extracted, as this was considered a surrogate indicator of multivessel coronary artery disease.

**Table 1:** Summary and baseline characteristics of the included studies

Trial, year	Region	Registration number	Population	Definition of multivessel disease	follow-up (months)	Arms	Patients, n	Age, mean ± SD	male, n (%)	Patients with multivessel disease, n (%)	Clinical Presentation			
											**Stable angina, n (%)**	**Unstable angina, n (%)**	**NSTEMI, n (%)**	**STEMI, n (%)**
IVUS-XPL 2020 [21]	Korea	NCT03866486	Patients who received DES to treat long coronary lesions	Two or three diseased vessels	60	IVUS	589	63 ± 9	408 (69)	375 (63.7)	291 (49)	211 (36)	_	_
						angiography	594	63 ± 9	409 (69)	490 (82.5)	307 (52)	189 (32)	_	_
ULTIMATE 2021 [22]	China	NCT02215915	All comers with de novo coronary lesions requiring DES implantation	_	36	IVUS	724	65.2 ± 10.9	535 (73.9)	381 (52.6)	_	_	_	_
						angiography	724	65.9 ± 9.8	530 (73.2)	414 (57.2)	_	_	_	_
RENOVATE-COMPLEX-PCI 2023 [23]	South Korea	NCT03381872	Patients with complex coronary-artery lesions undergoing PCI	At least two major epicardial coronary arteries are being treated at the same time	25.2	IVUS/OCT	1092	65.3 ± 10.3	869 (79.6)	409 (37.5)	532 (48.7)	361 (33.1)	171 (15.7)	28 (2.6)
						angiography	547	66.0 ± 10.0	431 (78.8)	213 (38.9)	275 (50.3)	173 (31.6)	87 (15.9)	12 (2.2)
OCTOBER 2023 [24]	Europe	NCT03171311	Patients with a clinical indication for PCI and a complex coronary artery bifurcation lesion	One or more additional target lesions treated either during the index procedure or at a staged procedure	24	OCT	600	66.4 ± 10.5	473 (78.8)	106 (17.7)	330 (55.0)	53 (8.8)	79 (13.2)	_
						angiography	601	66.2 ± 9.9	475 (79.0)	125 (20.8)	321 (53.4)	58 (9.7)	78 (13.0)	_
OCCUPI 2024 [25]	South Korea	NCT03625908	Patients requiring PCI with drug-eluting stents for one or more complex lesions	_	12	OCT	803	64 ± 10	646 (80)	203 (25.3)	391 (49)	248 (31)	118 (15)	46 (6)
						angiography	801	64 ± 9	644 (80)	212 (26)	423 (53)	215 (27)	105 (13)	58 (7)

*Abbreviations*: *DES* drug-eluting stent, *IVUS* intravascular ultrasound, *NCT* National Clinical Trial number, *NSTEMI* non–ST-segment elevation myocardial infarction, *OCT* optical coherence tomography, *PCI* percutaneous coronary intervention, *SD* standard deviation, *STEMI* ST-segment elevation myocardial infarction

### Outcome definitions

MACE was defined according to the definitions reported in each trial. Given the variability in MACE definitions across the included studies, the specific components of MACE as reported by each trial are summarized in Table S3.

In trials that did not explicitly report MACE as a composite outcome, target vessel failure (TVF) was extracted and used as a surrogate outcome, as its definition was consistent with the composite endpoints reported in other included studies, typically including cardiac death, target-vessel–related myocardial infarction, and clinically driven target-vessel revascularization. This approach was adopted to maximize data inclusion while maintaining clinical consistency across outcome definitions.

### Risk of bias assessment and certainty of evidence

The methodological quality and risk of bias for the included studies were assessed independently by the two reviewers (B.I. and T.Z.S.). RCTs were assessed for bias using the revised Cochrane Risk of Bias tool (RoB 2.0) [26], assessing bias across five domains: (1) randomization process, (2) deviations from intended interventions, (3) missing data of outcome, (4) measurement of outcome, and lastly (5) selection of the reported result. Classifying each domain into low risk, moderate risk, or high risk.

We assessed the certainty of evidence using the Grading of Recommendations Assessment, Development and Evaluation (GRADE) approach [27, 28].

### Data synthesis and statistical analysis

We conducted a pairwise meta-analysis comparing imaging-guided PCI with angiography-guided PCI using a random-effects model due to some definition heterogeneity across studies. Binary variables were pooled using the risk ratio (RR) and the Mantel–Haenszel method. Heterogeneity was quantified using I^2^, an I^2^ > 50% was classified as substantial heterogeneity. A *p*-value of < 0.1 was considered significant for heterogeneity, while a *p*-value of < 0.05 was considered significant for the pooled effect. Subsequent influence analysis and leave-one-out analysis were conducted to assess the robustness of the results and discover any influential studies that may skew the effect size or affect heterogeneity. A meta-regression was done using the follow-up period as a covariate to explore its association with the pooled effect size. Given the limited number of included studies, this analysis carries limited interpretability and should be interpreted as exploratory evidence.

Furthermore, to establish the robustness and conclusively of our results and guard against type I error resulting from repeated testing, we performed a trial sequential analysis (TSA). TSA calculates the required information size (RIS) beyond which adding more trials is unlikely to alter the effect size direction. For TSA, if the cumulative z-curve crossed either the trial sequential monitoring boundary (TSMB) or the RIS, the results are deemed conclusive. Otherwise, the result is considered inconclusive, and further evidence is required. For this analysis, we used a two-sided alpha level of 5%, a power of 80%, a relative risk reduction of 20%, and a model-based variance. We chose a 20% RRR as a conservative, clinically meaningful estimate to prevent over-optimistic results, where the RRR in the low-bias primary studies varied widely form 14.8% (RENOVATE-COMPLEX-PCI 2023) to 63.8% (OCCUPI 2024).

Additionally, to provide a greater clinical granularity and compare the three interventions (OCT, IVUS, and angiography) head-to-head, we performed an exploratory network meta-analysis (NMA). A frequentist NMA approach with a random effects model was used. P-score was calculated to rank the interventions; the P-score is a frequentist analog to the surface under the cumulative ranking curve (SUCRA) values, with higher P-scores indicating a higher probability that this intervention is superior to other interventions in the network. The same aforementioned cut-offs and significance levels were used.

All analyses were conducted using R software (Version 4.4.2, R Core Team) [29] except for TSA, which was performed using TSA software (Version 0.9.5.10 Beta, Copenhagen Trial Unit) [30].

Publication bias was not assessed due to the inclusion of fewer than 10 studies, as recommended by Egger et al. [31].

## Results

### Study selection and data extraction

**Fig. 1:**
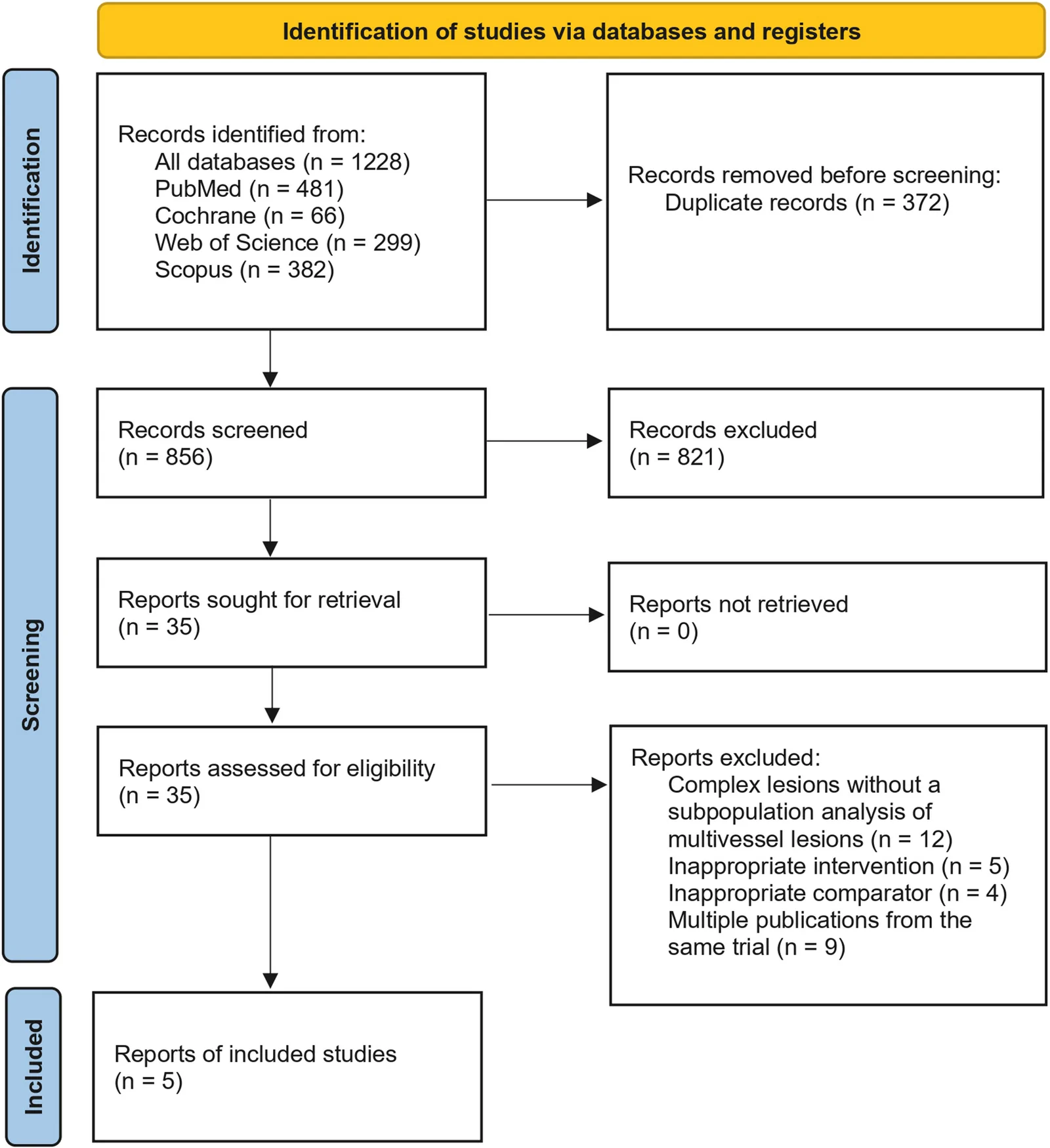
PRISMA flow diagram illustrating study identification, screening, eligibility assessment, and inclusion

A comprehensive database search revealed 1,228 studies. After removing duplicates, 856 articles were screened by title and abstract, and 35 were selected for full-text review. Among these, studies were excluded for the following reasons: complex lesions without a subpopulation analysis of multivessel lesions (*n* = 12), inappropriate interventions (*n* = 5), inappropriate comparators (*n* = 4), and multiple publications from the same trial (*n* = 9). Ultimately, five studies met the inclusion criteria for the meta-analysis. The study selection process is presented in Fig. 1. The MACE data of the included studies were extracted from the subpopulation analysis.

### Characteristics of the included studies

A total of five RCTs were included, comprising 3023 patients with multivessel coronary artery lesions. Among these, 1569 patients underwent intravascular imaging-guided PCI via either OCT or IVUS (OCT = 309; IVUS = 851; OCT/IVUS = 409, as some studies didn’t distinguish between the two), whereas 1454 patients underwent angiography-guided PCI. Among the intravascular imaging studies, 2 studies used IVUS exclusively, 2 studies used OCT exclusively, and only one study employed both IVUS and OCT. The summary and baseline characteristics of the included studies are presented in Table 1. The medical history characteristics of the included studies are presented in Table S4. The inclusion and exclusion criteria of each included study are presented in Table S5. The angiographic characteristics of the treated lesions across the included studies are presented in Table S6.

### Risk of bias assessment and certainty of evidence

**Fig. 2:**
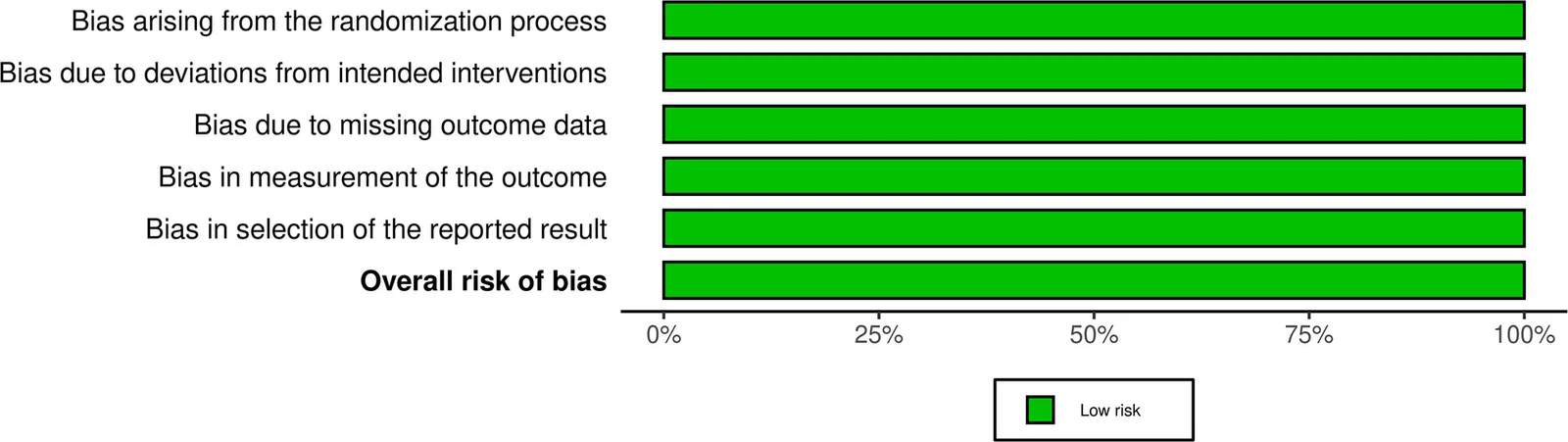
Summary of risk of bias assessment of included randomized controlled trials using the Cochrane RoB 2 tool

The five RCTs were judged to have a low risk of bias across all domains, as key methodological components, including random sequence generation, allocation concealment, and outcome assessment, were adequately reported in the included trials (Figs. 2 and S1). The certainty of evidence of total MACE outcome and each imaging modality subgrouping (IVUS and OCT) of MACE was rated as moderate certainty as the outcomes downgraded one level for indirectness due to reliance on subgroup-level data rather than primary randomized populations (Table 2).

**Table 2:** Summary of findings with quality of evidence

Outcomes	№ of participants (studies)	Anticipated absolute effects* (95% CI)		Relative effect (95% CI)	Certainty of the evidence (GRADE)
		Risk with Angiography	Risk with OCT or IVUS		
MACE (overall)	3023 (5 RCTs)	121 per 1,000	70 per 1,000 (56 to 90)	RR 0.58 (0.46 to 0.74)	⨁⨁⨁◯ Moderate^a^
MACE (IVUS-guided PCI)	1755 (2 RCTs)	117 per 1,000	62 per 1,000 (46 to 85)	RR 0.53 (0.39 to 0.73)	⨁⨁⨁◯ Moderate^a^
MACE (OCT-guided PCI)	646 (2 RCTs)	142 per 1,000	70 per 1,000 (39 to 124)	RR 0.49 (0.28 to 0.87)	⨁⨁⨁◯ Moderate^a^

Abbreviations: *MACE* major adverse cardiovascular events, *OCT* optical coherence tomography, *IVUS* intravascular ultrasound, *PCI* percutaneous coronary intervention, *RCTs* randomized controlled trials, *RR* risk ratio, *CI* confidence interval

^*^The risk in the intervention group (and its 95% confidence interval) is based on the assumed risk in the comparison group and the relative effect of the intervention (and its 95% CI)

^a^Downgraded for indirectness

### MACE

**Fig. 3:**
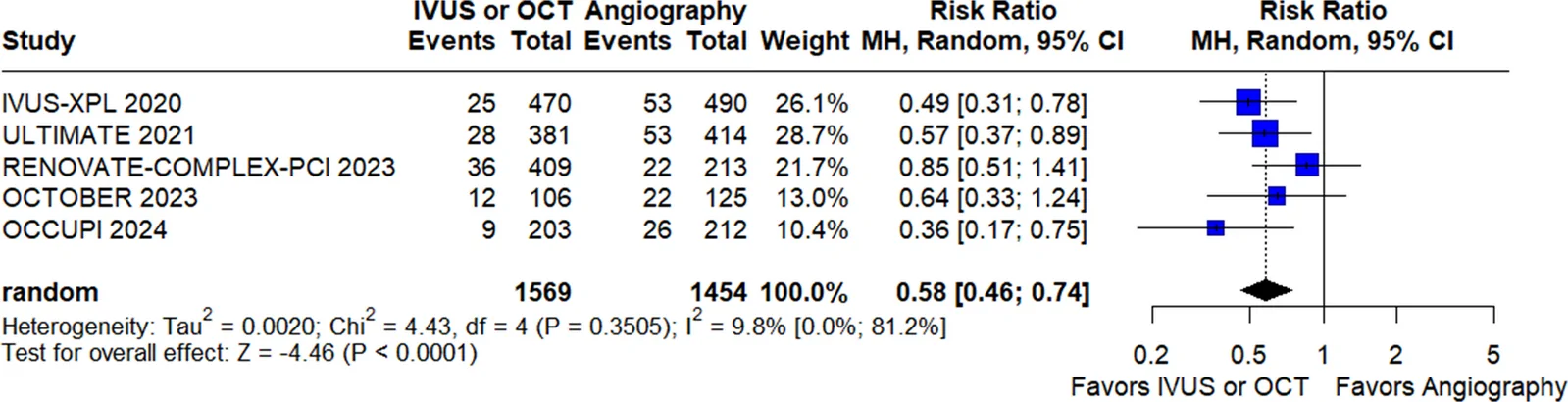
Forest plot comparing imaging-guided PCI (IVUS or OCT) versus angiography-guided PCI for MACE

Imaging-guided PCI showed a statistically significant association with decreased MACE rates compared to angiography-guided PCI (RR: 0.58, 95% CI [0.46, 0.74]; *p* < 0.001) (Fig. 3) corresponding to an absolute risk reduction of 51 events per 1,000 patients. No heterogeneity was detected across the studies (I^2^ = 9.8%, 95% CI [0.0, 81.2], *p* = 0.35).

**Fig. 4:**
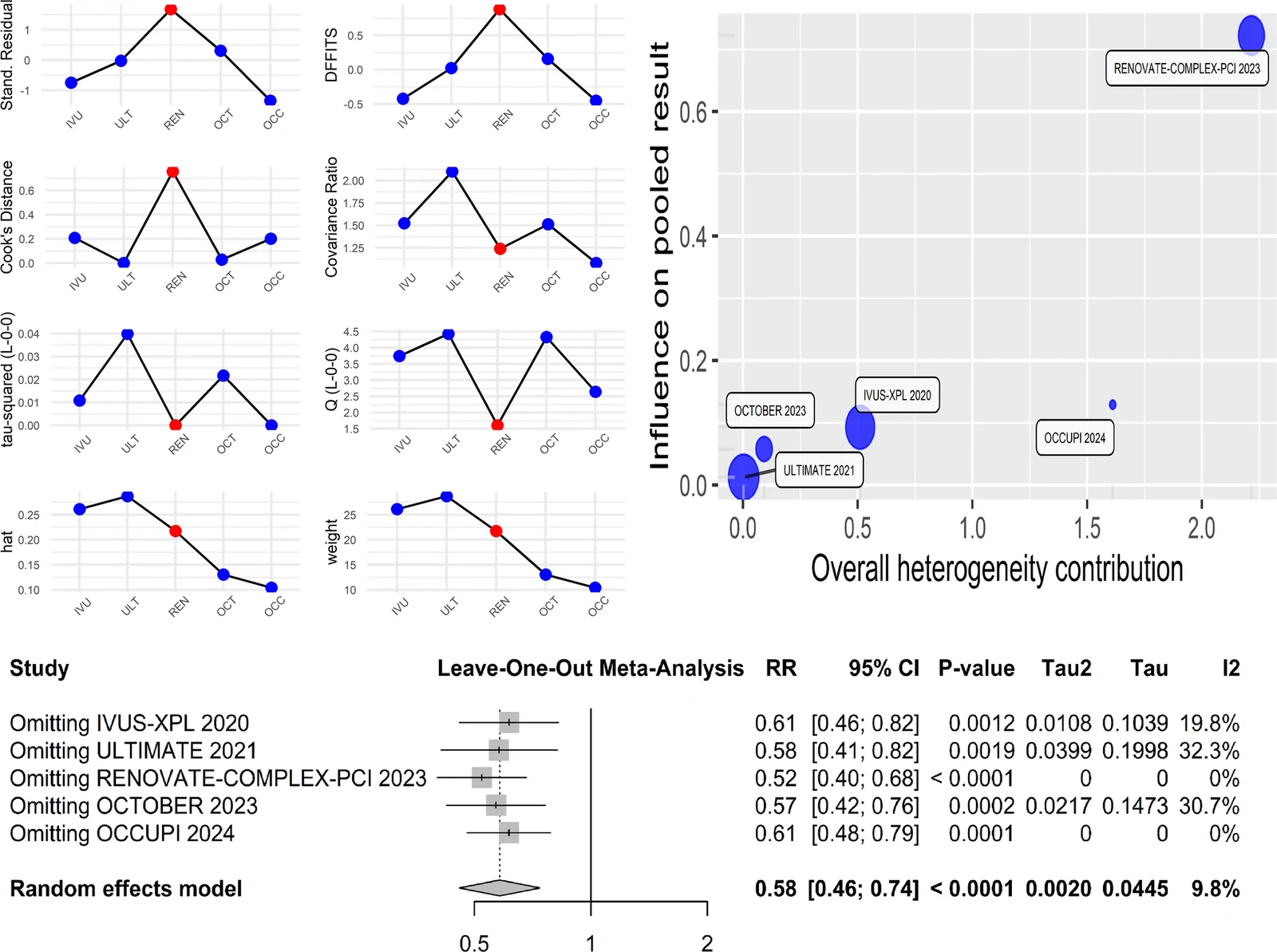
Influence, diagnostic plots and leave-one-out analysis evaluating the impact of individual studies on the pooled effect estimate and heterogeneity

The influence analysis indicated that the RENOVATE-COMPLEX-PCI 2023 trial [23] was an influential study (Fig. 4). Upon its exclusion, heterogeneity dropped to (I^2^ = 0.0%, 95% CI [0.0; 84.7], *p* = 0.66), and the effect size remained significant (RR: 0.52, 95% CI [0.40; 0.68], *p* < 0.001) (Figure S2).

Meta-regression demonstrated that the follow-up time of the studies was not a statistically significant Moderator of the effect (*p* = 0.47, *R*^2^ = 0.0%).

**Fig. 5:**
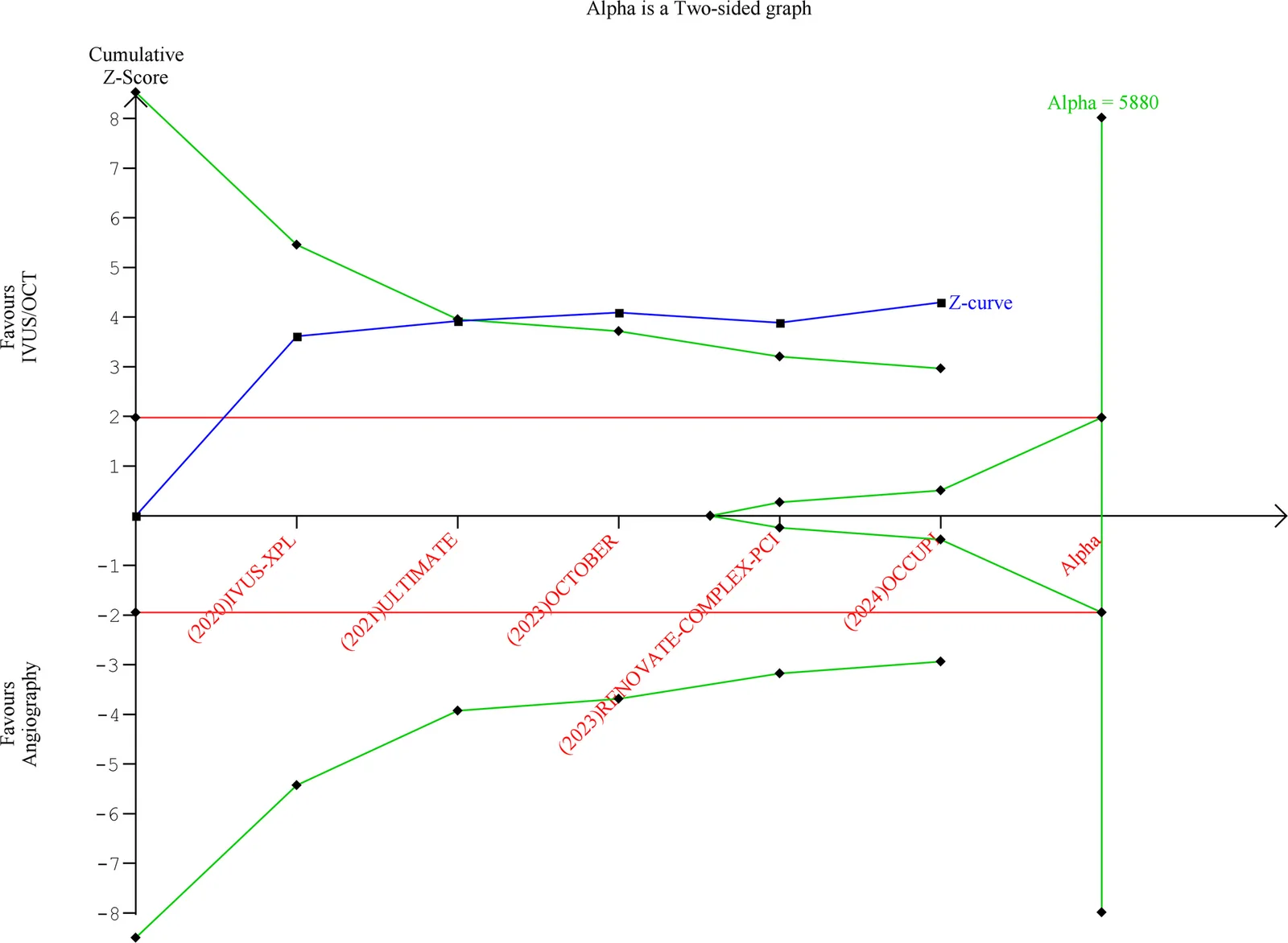
TSA for MACE comparing imaging-guided PCI versus angiography-guided PCI. Trial sequential analysis for MACE of intravascular imaging compared with angiography-guided PCI was performed via a random-effects model to evaluate the conclusiveness of the evidence for a relative risk of 0.58 (CI: 0.46 to 0.74). A required information size of 5880 was calculated based on a 20% RRR, with a double-sided Alpha of 0.05, a power of 80.0%, and inconsistency (I^2^) and (D^2^) of 10% and 12%, respectively. The cumulative Z-curve (blue line) crossed the superiority boundary; it did not reach the RIS sample line, which suggests that the cumulative evidence supports that intravascular imaging is superior to angiography-guided PCI, given the existing evidence

TSA showed that the cumulative z-curve for MACE crosses the TSMB of benefit, favoring imaging; however, it did not reach the RIS (Fig. 5) with an alpha-spending adjusted CI of 0.40 to 0.85. These findings indicate that the current evidence supports the beneficial effect of imaging on MACE, although the RIS was not fully reached, suggesting that further trials may be needed.

In subgroup analyses according to imaging modality, IVUS-guided PCI was associated with a significant reduction in MACE compared with angiography (RR 0.53, 95% CI [0.39;0.73], *p* < 0.0001; I^2^ = 0%), as was OCT-guided PCI (RR 0.49, 95% CI [0.28–0.87], *p* = 0.014; I^2^ = 24.3%) (Figure S3).

**Fig. 6:**
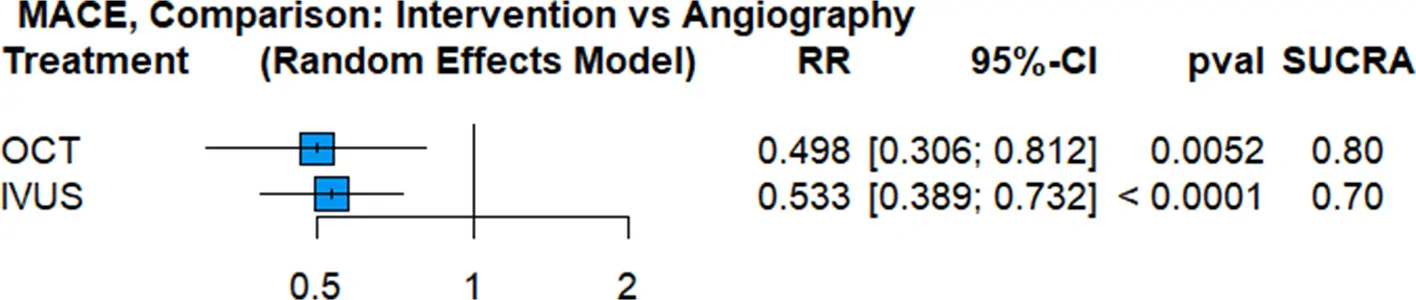
NMA comparing IVUS- and OCT-guided PCI versus angiography for MACE

The exploratory NMA for MACE comprised four studies, with two comparing IVUS with angiography and two comparing OCT with angiography (Figs. 6, S4, and S5). The NMA demonstrated that both IVUS and OCT were superior to angiography (RR: 0.53, 95% CI [0.39, 0.73], *p* < 0.001, and RR: 0.50, 95% CI [0.31, 0.81], *p* = 0.005), respectively. While there was no significant difference between IVUS vs. OCT in the indirect comparison (RR: 1.07, 95% CI [0.60, 1.91], *p* = 0.82), p-scores were higher for OCT (0.79 vs. 0.71), respectively. There were no closed loops in this network; therefore, a formal consistency assessment was not applicable. Additionally, due to the small number of studies and the lack of direct IVUS vs OCT comparison, treatment rankings derived from this NMA should be interpreted cautiously and considered exploratory rather than definitive.

## Discussion

In this systematic review and meta-analysis, incorporated with TSA and exploratory NMA, we evaluated the use of intravascular imaging-guided PCI compared to conventional angiography-guided PCI in patients with multivessel coronary artery disease regarding MACE. Our findings demonstrated that imaging-guided PCI is associated with a significant 42% relative risk reduction in MACE, with confirmed evidence that supports the superiority of imaging-guided PCI and with a high outcome certainty. Also, our results showed that both IVUS and OCT are superior to angiography, with no difference between the two imaging modalities. The overall findings are based on randomized comparisons and provide supportive evidence, whereas subgroup and network meta-analysis findings should be interpreted as exploratory.

While prior studies and recent meta-analyses have evaluated intravascular imaging in broader complex PCI populations, these cohorts often included heterogeneous lesion subsets. In contrast, the present analysis specifically focuses on patients with multivessel coronary artery disease, providing a more targeted assessment within this clinically relevant subgroup.

Several mechanisms may explain the observed benefit of imaging-guided PCI. Intravascular imaging enables accurate vessel sizing and optimal stent expansion. It also facilitates detection of complications such as malapposition or edge dissections, which may reduce adverse events such as restenosis and stent thrombosis [9, 11].

In addition, emerging evidence suggests that the benefits of intravascular imaging may be particularly pronounced in high-risk lesion subsets. For example, recent data have highlighted the role of IVUS guidance in improving outcomes in isolated ostial left anterior descending artery lesions, underscoring the value of imaging in anatomically complex and prognostically significant coronary segments [32].

Beyond trial-level efficacy, the clinical relevance of imaging-guided PCI lies in improving procedural decision-making in high-risk coronary artery disease. Large contemporary observational data from patients with multivessel coronary artery disease presenting with acute myocardial infarction and cardiogenic shock have demonstrated substantial variability in revascularization practices and outcomes, highlighting ongoing uncertainty in optimal procedural strategies at the health-system level [33]. In this context, emerging functional assessment tools such as quantitative flow ratio have been proposed to improve physiological lesion assessment and guide revascularization strategies [34]. These findings support a more prominent role for intravascular imaging in contemporary PCI practice, particularly in patients with complex or multivessel coronary artery disease, and are consistent with evolving guideline recommendations.

Current guideline recommendations for imaging-guided PCI in multivessel coronary artery disease remain limited. The 2021 ACC/AHA/SCAI Guideline considers the use of intravascular imaging reasonable (Class IIa) to optimize PCI in anatomically complex settings, reflecting higher procedural risk and lesion complexity, although specific recommendations for multivessel disease are not explicitly explained [16]. In this context, our findings provide randomized evidence demonstrating a significant reduction in MACE with imaging-guided PCI in patients with multivessel coronary artery disease, supporting a more prominent role for intravascular imaging in this high-risk population.

Our findings in patients with multivessel coronary artery disease are consistent with prior randomized trials evaluating intravascular imaging-guided PCI in broader populations of complex coronary lesions. The IVUS-XPL trial demonstrated a sustained reduction in MACE with IVUS-guided PCI compared with angiography-guided PCI in patients with long and complex lesions over a 5-year follow-up [21]. Similarly, the OCTOBER trial showed that OCT-guided PCI significantly reduced MACE at 2 years in patients with complex bifurcation lesions [24]. While the OCCUPI trial reported a lower incidence of MACE at 1 year among patients undergoing OCT-guided PCI for overall complex coronary lesions [25]. Although these trials primarily enrolled heterogeneous complex-lesion populations, the concordant direction and magnitude of benefit observed in our multivessel disease subgroup analysis support the external validity of imaging guidance in this particularly high-risk cohort.

In concordance with our results, a comprehensive meta-analysis by Ahmed et al., 2025 based on randomized trials in a heterogeneous PCI population, demonstrated a lower risk of MACE with IVUS-guided PCI compared with angiography guidance [35]. In contrast, a contemporary meta-analysis by Sreenivasan et al., 2024 which specifically evaluated complex lesion subsets, reported a consistent reduction in MACE with intravascular imaging-guided PCI [13].

Although the majority of clinical trials and systematic reviews are in agreement with our results, they evaluated the complex lesions and heterogeneous PCI populations, and there remains a lack of systematic reviews that focused exclusively on the subgroup of multivessel coronary artery disease.

This study focuses on multivessel coronary artery disease, a high-risk population often underrepresented in prior analyses. High-quality evidence from randomized trials involving 3,023 patients, supported by conclusive TSA and exploratory network meta-analysis, strengthens the reliability of the findings.

### Limitations

Several limitations of this study should be acknowledged. First, outcome extraction was derived from subgroup data of the included clinical trials, which may introduce indirectness and limit the robustness of pooled estimates. Although subgroup-level data were used, these analyses were derived from randomized comparisons within the included trials, preserving the internal validity of treatment effect estimates. Therefore, while indirectness was considered, it was not deemed sufficient to warrant downgrading the certainty of evidence. However, all included studies were rated as low risk of bias, and we conducted TSA, which showed that the cumulative evidence supports a beneficial effect, although the RIS was not fully reached, supporting the overall reliability of our findings. Second, there was variability in the definition of MACE across the included trials, which may affect the comparability of pooled outcomes; detailed definitions for each trial are provided in Table S3. In addition, limited reporting of individual MACE components precluded a detailed analysis of which specific endpoints primarily drove the observed benefit of imaging-guided PCI. Third, differences in sample size across the included studies resulted in unequal study weights within the meta-analysis, potentially influencing the pooled effect estimates. Fourth, meta-regression was performed to explore the impact of follow-up duration; the analysis yielded an *R*^2^ value of 0.0%, limiting the interpretability of the regression graph. Fifth, there was variability in the duration and timing of follow-up across trials, which may contribute to clinical heterogeneity. Additionally, follow-up durations varied across the included trials and are detailed in Table 1. Given the limited number of studies, further analyses to explore differences between short- and long-term outcomes were not feasible. Furthermore, inconsistent and incomplete reporting of multivessel coronary artery disease definitions across studies represents an additional potential source of clinical variability. Additionally, the exploratory network meta-analysis was limited by the absence of closed loops, which precluded formal consistency assessment and necessitates cautious interpretation of indirect comparisons between IVUS and OCT. Finally, publication bias was not formally assessed, as the number of included studies was fewer than ten, limiting the reliability of funnel plot-based or statistical assessments.

### Future research directions

Future research should focus on conducting adequately powered RCTs specifically designed to evaluate intravascular imaging-guided PCI in patients with multivessel coronary artery disease as a distinct population rather than a subgroup of complex lesions. Standardization of definitions for multivessel disease and MACE components is warranted to improve comparability across studies. In addition, future trials should report individual MACE components separately to clarify which outcomes primarily drive the observed clinical benefit. Longer and more uniform follow-up durations are needed to assess the durability of imaging-guided PCI benefits. Further studies comparing IVUS and OCT head-to-head in multivessel disease populations, as well as cost-effectiveness analyses, would provide valuable insights to inform clinical practice and guideline recommendations.

## Conclusion

Multivessel coronary artery disease represents a high-risk clinical setting in which optimal procedural guidance is essential. In this systematic review and meta-analysis, the available evidence suggests that intravascular imaging-guided PCI is associated with a reduction in MACE compared with angiography guidance alone. Incorporating IVUS or OCT into PCI decision-making may help optimize stent deployment and may improve long-term patient outcomes in this population. Future research should focus on dedicated randomized trials in multivessel disease to further refine patient selection, compare imaging modalities, and inform future guideline recommendations.

## Supplementary Information

Supplementary Material 1.

## Data Availability

All data used in this study are publicly available from the included studies.

## Abbreviations

ACC: American College of Cardiology
ACS: Acute Coronary Syndromes
AHA: American Heart Association
CI: Confidence Interval
DES: Drug-Eluting Stent
GRADE: Grading of Recommendations Assessment, Development and Evaluation
IVUS: Intravascular Ultrasound
MACE: Major Adverse Cardiovascular Events
MeSH: Medical Subject Headings
NMA: Network Meta-Analysis
OCT: Optical Coherence Tomography
PCI: Percutaneous Coronary Intervention
PRISMA: Preferred Reporting Items for Systematic Reviews and Meta-Analyses
RCT: Randomized Controlled Trial
RIS: Required Information Size
RR: Risk Ratio
SD: Standard Deviation
SUCRA: Surface Under the Cumulative Ranking Curve
TSA: Trial Sequential Analysis
TSMB: Trial Sequential Monitoring Boundary
TVF: Target Vessel Failure
