# Supplementary material for: Intravascular imaging-guided versus angiography-guided percutaneous coronary intervention in multivessel coronary artery disease: a systematic review, meta-analysis, and trial sequential analysis of randomized controlled trials
Source: BMC Cardiovasc Disord. 2026 Jun 15;26:729. doi: 10.1186/s12872-026-06135-7 (PMC13495394; doi:10.1186/s12872-026-06135-7)
Supplement: Supplementary file 1 [file 12872_2026_6135_MOESM1_ESM.docx]

**Supplementary Appendix**

***Supplement to: Intravascular Imaging-Guided Versus Angiography-Guided Percutaneous Coronary Intervention in Multivessel Coronary Artery Disease: A Systematic Review, Meta-Analysis, and Trial Sequential Analysis of Randomized Controlled Trials***

**Table of Contents**

|  | | Page |
| --- | --- | --- |
| Supplemental Figure 1. | Traffic light plot summarizing the risk of bias across included studies | 2 |
| Supplemental Figure 2. | Forest plot of the sensitivity analysis comparing imaging-guided PCI (IVUS or OCT) versus angiography-guided PCI for MACE, excluding RENOVATE-COMPEX-PCI 2023 trial. | 3 |
| Supplemental Figure 3. | Forest plot of subgroup analysis by imaging modality used, comparing imaging-guided PCI (IVUS or OCT) versus angiography-guided PCI for MACE. | 4 |
| Supplemental Figure 4. | Network plot showing direct comparisons between angiography-, IVUS-, and OCT-guided PCI of MACE. | 5 |
| Supplemental Figure 5. | NMA comparing IVUS- and angiography-guided PCI versus OCT for MACE. | 6 |
| Supplemental Table 1. | Trial acronyms and corresponding full trial names | 7 |
| Supplemental Table 2. | Summary of the search strategy for all the databases | 8 |
| Supplemental Table 3. | Definitions of composite clinical outcomes (MACE and its corresponding TVF) across included trials | 9 |
| Supplemental Table 4. | Baseline medical history characteristics of the included trials. | 10 |
| Supplemental Table 5. | Inclusion and exclusion criteria of the included trials. | 11 |
| Supplemental Table 6. | Baseline angiographic characteristics of the included trials. | 12 |

**Supplementary Figure 1:** Traffic light plot summarizing the risk of bias across included studies.

**Supplementary Figure 2:** Forest plot of the sensitivity analysis comparing imaging-guided PCI (IVUS or OCT) versus angiography-guided PCI for MACE, excluding RENOVATE-COMPEX-PCI 2023 trial.

**Supplementary Figure 3:** Forest plot of subgroup analysis by imaging modality used, comparing imaging-guided PCI (IVUS or OCT) versus angiography-guided PCI for MACE.

**Supplementary Figure 4:** Network plot showing direct comparisons between angiography-, IVUS-, and OCT-guided PCI of MACE.

**Supplementary Figure 5:** NMA comparing IVUS- and angiography-guided PCI versus OCT for MACE.

| **Trial (Year)** | **Full trial name** |
| --- | --- |
| IVUS-XPL 2020 | Impact of Intravascular Ultrasound Guidance on the Outcomes of Xience Prime Stents in Long Lesions |
| ULTIMATE 2021 | Intravascular Ultrasound Guided Drug-Eluting Stents Implantation in “All-Comers” Coronary Lesions |
| RENOVATE-COMPLEX-PCI 2023 | Randomized Evaluation of Intravascular Imaging–Guided versus Angiography-Guided Complex Percutaneous Coronary Intervention |
| OCTOBER 2023 | Optical Coherence Tomography or Angiography Guidance for PCI in Complex Bifurcation Lesions |
| OCCUPI 2024 | Optical Coherence Tomography–Guided versus Angiography-Guided Percutaneous Coronary Intervention for Complex Lesions |

**Supplementary Table 1:** Trial acronyms and corresponding full trial names.

**Supplementary Table 2:** Summary of the search strategy for all the databases.

| **Search string** | (“Intravascular Imaging” OR “coronary imaging” OR “vascular imaging” OR “intravascular ultrasound” OR “IVUS” OR “vascular ultrasound” OR “IVUS-guided PCI” OR “optical coherence tomography” OR “OCT” OR “angioscopy” OR “intravascular fluoroscopy”) AND (“coronary angiography” OR “cardiac angiography” OR “angiography” OR “coronary arteriography” OR “cardiac catheterization”) AND (“multivessel disease” OR “multivessel coronary disease” OR “multiple vessel disease” OR “two-vessel disease” OR “three-vessel disease” OR “diffuse coronary disease” OR “complex lesion” OR “complex coronary lesions”) | | |
| --- | --- | --- | --- |
| **Database** | PubMed | 481 | No filter |
|  | Scopus | 382 | No filter |
|  | Web of Science | 299 | No filter |
|  | Cochrane CENTRAL | 66 | No filter |
| **Date of search** | 16/12/2025 | | |

**Supplementary Table 3:** Definitions of composite clinical outcomes (MACE and its corresponding TVF) across included trials.

| **Trial, year** | **Outcome definition** |
| --- | --- |
| IVUS-XPL 2020 **[31]** | **MACE** Cardiac death, target lesion-related myocardial infarction, or ischemia-driven target lesion |
| ULTIMATE 2021 **[35]** | **TVF** Cardiac death, TVMI, and clinically driven TVR |
| RENOVATE-COMPLEX-PCI 2023 **[27]** | **TVF** Death from cardiac causes, target-vessel–related myocardial infarction, or clinically driven target-vessel revascularization |
| OCTOBER 2023 **[32]** | **MACE** Death from a cardiac cause, target-lesion MI, or ischemia-driven target-lesion revascularization. |
| OCCUPI 2024 **[33]** | **MACE** Composite of cardiac death, myocardial infarction, stent thrombosis, or ischemia-driven target-vessel revascularization. |

**Supplementary Table 4:** Baseline medical history characteristics of the included trials.

| **Trial, year** | **Arms** | **Medical History** | | | | | | | | | |
| --- | --- | --- | --- | --- | --- | --- | --- | --- | --- | --- | --- |
|  |  | **BMI, mean ± SD** | **Hyperlipidemia, n (%)** | **CKD, n (%)** | **MI, n (%)** | **DM, n (%)** | **Current smoking, n (%)** | **CABG, n (%)** | **Stroke, (%)** | **PCI, n (%)** | **HTN, n (%)** |
| IVUS-XPL 2020 **[31]** | IVUS | 24.7 ±2.9 | 396 (67) | _ | 30 (5) | 178 (30) | 134 (23) | 16 (3) | _ | 66 (11) | 382 (65) |
|  | angiography | 24.8 ± 3.0 | 393 (66) | _ | 27 (5) | 206 (35) | 153 (26) | 16 (3) | _ | 60 (10) | 373 (63) |
| ULTIMATE 2021 **[35]** | IVUS | _ | 389 (53.7) | 180 (24.9) | _ | 217 (30.0) | _ | _ | _ | _ | 512 (70.7) |
|  | angiography | _ | 400 (55.2) | 169 (23.3) | _ | 226 (31.2) | _ | _ | _ | _ | 521 (72.0) |
| RENOVATE-COMPLEX-PCI 2023 **[27]** | IVUS/OCT | _ | 560 (51.3) | 203 (18.6) | 75 (6.9) | 394 (36.1) | 212 (19.4) | _ | 70 (6.4) | 268 (24.5) | 682 (62.5) |
|  | angiography | _ | 280 (51.2) | 93 (17.0) | 42 (7.7) | 223 (40.8) | 95 (17.4) | _ | 42 (7.7) | 127 (23.2) | 323 (59.0) |
| OCTOBER 2023 **[32]** | OCT | 28.0 ± 4.6 | 456 (76.0) | 12 (2.0) | 170 (28.3) | 103 (17.2) | 77 (12.8) | _ | 15 (2.5) | 244 (40.7) | 422 (70.3) |
|  | angiography | 28.2 ± 4.9 | 471 (78.4) | 14 (2.3) | 180 (30.0) | 97 (16.1) | 85 (14.1) | _ | 14 (2.3) | 257 (42.8) | 448 (74.5) |
| OCCUPI 2024 **[33]** | OCT | 24.8 ± 2.7 | 684 (85) | 53 (7) | 40 (5) | 261 (33) | 149 (19) | 10 (1) | 38 (5) | 171 (21) | 466 (58) |
|  | angiography | 24.7 ± 2.7 | 661 (83) | 57 (7) | 42 (5) | 262 (33) | 158 (20) | 14 (2) | 46 (6) | 159 (20) | 451 (56) |

BMI = body mass index; CABG = coronary artery bypass grafting; CKD = chronic kidney disease; DM = diabetes mellitus; HTN = hypertension; IVUS = intravascular ultrasound; MI = myocardial infarction; n = number; OCT = optical coherence tomography; PCI = percutaneous coronary intervention; SD = standard deviation.

| **Trial, year** | **Inclusion Criteria** | **Exclusion Criteria** |
| --- | --- | --- |
| IVUS-XPL 2020 **[31]** | • Age ≥20 years  • Typical angina or objective evidence of myocardial ischemia  • De novo coronary lesion suitable for PCI  • Reference vessel diameter 2.5–4.0 mm (operator assessment) | • ST-segment elevation myocardial infarction (STEMI) within 48 h  • Cardiogenic shock  • Left main disease requiring surgery  • Chronic total occlusion  • Previously implanted drug-eluting stent within 6 months  • In-stent restenosis |
| ULTIMATE 2021 **[35]** | • Men or women aged ≥18 years  • Indication for PCI according to current guidelines  • Target lesion suitable for drug-eluting stent implantation and IVUS imaging | • Pregnancy or breastfeeding  • Severe comorbidities limiting life expectancy  • STEMI within 24 h from symptom onset |
| RENOVATE-COMPLEX-PCI 2023 **[27]** | • Age ≥19 years  • Coronary artery disease requiring complex PCI  • Ability to provide written informed consent | • Target lesions not suitable for PCI (operator decision)  • Contraindication to antiplatelet therapy  • Severe comorbidities judged by the investigator  • Inability or unwillingness to comply with follow-up |
| OCTOBER 2023 **[32]** | • Stable angina, unstable angina, or clinically stabilized acute coronary syndrome  • Bifurcation lesions suitable for provisional or two-stent techniques | • STEMI within 72 h  • Cardiogenic shock  • Prior coronary artery bypass grafting of target vessel  • Left main coronary artery thrombus  • Medina 0,0,1 lesions |
| OCCUPI 2024 **[33]** | • Age 19–85 years  • Ischemic heart disease (including stable angina or ACS)  • De novo coronary lesions suitable for PCI | • Severe hepatic dysfunction (≥3× upper normal limit)  • Severe renal dysfunction or dialysis  • Life expectancy <1 year  • Stent thrombosis or in-stent restenosis  • Bypass graft lesions  • Inability to understand informed consent |

**Supplementary Table 5:** Inclusion and exclusion criteria of the included trials.
ACS = acute coronary syndrome; CABG = coronary artery bypass grafting; DES = drug-eluting stent; IVUS = intravascular ultrasound; LAD = left anterior descending artery; LCX = left circumflex artery; MI = myocardial infarction; OCT = optical coherence tomography; PCI = percutaneous coronary intervention; RCA = right coronary artery; STEMI = ST-segment elevation myocardial infarction.

**Supplementary Table 6:** Baseline angiographic characteristics of the included trials.

| **Trial, year** | **Arms** | **Patients with multivessel disease, n (%)** | **Baseline Quantitative Coronary Angiographic Data** | | | | | | | |
| --- | --- | --- | --- | --- | --- | --- | --- | --- | --- | --- |
|  |  |  | **MLD (mm)** | **Type of stent used** | **Lesion length (mm)** | **Mean stent diameter + SD** | **Total Lesion number** | **LAD, n (%)** | **LCX, n (%)** | **RCA, n (%)** |
| IVUS-XPL 2020 **[31]** | IVUS | 375 (63.7) | 0.83 ± 0.43 | everolimus-eluting stent | 35.1 ± 10.8 | _ | _ | 393 (67) | 77 (13) | 119 (20) |
|  | angiography | 490 (82.5) | 0.81 ±0.43 | everolimus-eluting stent | 35.3 ± 10.7 | _ | _ | 358 (60) | 92 (16) | 144 (24) |
| ULTIMATE 2021 **[35]** | IVUS | 381 (52.6) | _ | Second-generation DES | _ | 3.15 ± 0.42 | 962 | 552 (57.4) | _ | _ |
|  | angiography | 414 (57.2) | _ | Second-generation DES | _ | 2.99 ± 0.38 | 1,016 | 561 (55.2) | _ | _ |
| RENOVATE-COMPLEX-PCI 2023 **[27]** | IVUS/OCT | 409 (37.5) | 0.44 ± 0.37 | Biodegradable or biocompatible polymer-coated everolimus-eluting stents | 28.4 ± 15.9 | 3.1 ± 0.5 | _ | 701 (43.2) | 313 (19.3) | 445 (27.4) |
|  | angiography | 213 (38.9) | 0.44 ± 0.36 | Biodegradable or biocompatible polymer-coated everolimus-eluting stents | 26.8 ± 14.8 | 3.0 ± 0.4 | _ | 376 (46.1) | 151 (18.5) | 215 (26.4) |
| OCTOBER 2023 **[32]** | OCT | 106 (17.7) | _ | Xience DES /Other DES | _ | _ | _ | _ | _ | _ |
|  | angiography | 125 (20.8) | _ | Xience DES /Other DES | _ | _ | _ | _ | _ | _ |
| OCCUPI 2024 **[33]** | OCT | 203 (25.3) | 0.7 ± 0.34 | everolimus-eluting stents | 35.5 ± 18 | 3.2 ± 0.37 | 944 | _ | _ | _ |
|  | angiography | 212 (26) | 0.7 ± 0.36 | everolimus-eluting stents | 34 ± 17.5 | 3.1 ± 0.37 | 950 | _ | _ | _ |

DES = drug-eluting stent; IVUS = intravascular ultrasound; LAD = left anterior descending artery; LCX = left circumflex artery; MLD = minimal lumen diameter; n = number; OCT = optical coherence tomography; PCI = percutaneous coronary intervention; RCA = right coronary artery; RVD = reference vessel diameter; SD = standard deviation.
